# Change in health and social factors in mid-adulthood and corresponding changes in leisure-time physical inactivity in a prospective cohort

**DOI:** 10.1186/s12966-018-0723-z

**Published:** 2018-09-15

**Authors:** Snehal M. Pinto Pereira, Chris Power

**Affiliations:** 1 MRC Unit for Lifelong Health and Ageing at UCL, 33 Bedford Place, London, WC1B 5JU UK; 2 Population, Policy and Practice, UCL Great Ormond Street Hospital Institute of Child Health, University College London, London, WC1N 1EH UK

**Keywords:** Birth cohort, Britain, Leisure-time physical inactivity, Life-course

## Abstract

**Background:**

To identify whether changes in adult health and social factors are associated with simultaneous changes in inactivity.

**Methods:**

Health, social factors and leisure-time inactivity (activity frequency < 1/week) were self-reported at 33y and 50y in the 1958 British birth cohort (*N* = 12,271). Baseline (33y) health and social factors and also patterns of change in factors 33y-to-50y were related to inactivity 33y-to-50y (never inactive, persistently inactive, deteriorating to inactivity, or improving from inactivity) using multinomial logistic regression.

**Results:**

Approximately 31% were inactive at 33y and 50y; 35% changed status 33y-to-50y (17% deteriorating to inactivity, 18% improving from inactivity). Baseline poor health and obesity were associated with subsequent (33y-to-50y) inactivity; e.g. for poor health, relative risk ratios (RRRs) for deteriorating to inactivity (vs never inactive) and improving from inactivity (vs persistently inactive) were 1.38(1.16,1.64) and 0.77(0.63,0.94) respectively. Adverse changes in health and weight were associated with simultaneous adverse changes in inactivity; e.g. worsening health (vs always good/excellent health) was associated with higher risk of deteriorating to inactivity (RRR:2.20(1.85,2.62)) and lower risk of improving from inactivity (RRR:0.61(0.49,0.77)). However, improving health and weight loss were not associated with improving from inactivity. Worsening self-efficacy 33y-to-50y was associated with lower risk of improving from inactivity; there was no association between improving self-efficacy and inactivity change. Downward social mobility was not associated with deteriorating to or improving from inactivity. Changes in depression symptom level, marriage/co-habitation or parenthood 33y-to-50y were not associated with inactivity changes. No associations were observed for employment.

**Conclusions:**

Associated changes in mid-life health factors with deleterious inactivity changes, highlight the importance of maintaining health, weight and self-efficacy across adulthood to deter inactivity.

**Electronic supplementary material:**

The online version of this article (10.1186/s12966-018-0723-z) contains supplementary material, which is available to authorized users.

## Background

In the UK, 44% of adults never do any moderate intensity physical activity [1] and globally, 23% are inactive [2] (i.e. do not meet physical activity recommendations). The latter is of concern because inactivity has high health and economic burdens [3–5]; inactivity prevention is therefore an important public health goal [6]. However, knowledge gaps relating to inactivity need addressing, and one such gap concerns influences on changes in inactivity behaviours. Physical inactivity tracks at low to moderate levels in adulthood [7], and understanding is needed on why some people continue to have a stable behaviour (i.e. be physically active or inactive over time) or why their behaviour changes (i.e. deteriorates to or improves from inactivity). Such knowledge would contribute evidence relevant to the planning of public health interventions.

Several correlates of physical inactivity have been identified [8] and studies have examined circumstances at baseline with inactivity several years later [9, 10]. However, while some longitudinal studies have examined concurrent influences on inactivity [11, 12], with few exceptions [13], the impact of change in factors on inactivity patterns is unclear. Likely influences on inactivity include poor health [9], adiposity [14], depression [15], low self-efficacy [16] and social factors such as socio-economic position (SEP) [17] and parenthood [18]. Physical factors (e.g. poor health and adiposity) could hinder activity via dyspnea [14] or other functional limitations. Factors related to mental functioning (e.g. depression and low self-efficacy) could operate via outcome expectations; e.g. low self-efficacy may reduce expectations about what physical activity will achieve [16]. Social factors (e.g. manual SEP) could limit the availability of, or a person’s response to, opportunities for activity [19]. Such health and social factors are dynamic and subject to change as individuals’ age, yet, these changes are usually ignored. To illustrate, relationships between poor health and subsequent inactivity have been examined [9], but little attention has been given to simultaneous changes in health and inactivity. Patterns over time are important to consider, because when a factor remains largely stable, examining associations between the factor at baseline with change in inactivity could potentially lead to null findings [20]; e.g. change in SEP may be more relevant to inactivity change than stable SEP. Thus, acknowledging the limited available evidence, calls have been made for longitudinal studies to assess the impact of changes in factors on changes in physical inactivity [10, 20, 21].

Therefore, using a nationwide general population sample, we aimed to identify associations of potential contemporary health (poor health, adiposity, depression, low self-efficacy) and social (manual SEP, not in paid employment, marriage/co-habitation, parenthood) factors with adult leisure-time inactivity. Specific objectives were to examine associations of (i) baseline factors at 33y with subsequent inactivity 33y-to-50y and (ii) patterns of change in factors 33y-to-50y with inactivity over the same ages. Previous work by ourselves [7] and others [17, 22] suggests that research on adult inactivity should consider influences on inactivity from earlier life-stages, hence we assessed whether associations remained after taking account of early-life factors. Given the lack of a consistent definition, we, like others [23–26], defined inactivity as activity frequency < 1/week, which is associated with increased risk of unfavourable outcomes [24–26], including mortality [24, 25].

## Methods

The 1958 British Birth Cohort is an ongoing longitudinal study of all born during one week in March 1958 across England, Scotland and Wales (*N* = 17,638) and a further 920 immigrants with the same birth week [27]. Information was collected in childhood (birth, 7, 11 and 16y) and adulthood (23, 33, 42, 45 and 50y). Ethical approval was given, including at 50y by the London Multi-centre Research Ethics Committee; informed consent was obtained from participants at various ages. Respondents in mid-adulthood are broadly representative of the surviving cohort [28]; this study consists of those living in Britain at 50y with information on inactivity at either 33y or 50y (*N* = 12,271).

*Physical inactivity at 33y and 50y* was ascertained using the same questions, asking participants about regular leisure-time activity frequency. In the questionnaire, ‘regular’ was defined as ≥1/month for most of the year and, to aid recall, a list of example activities (of predominantly moderate or vigorous intensity [29] e.g. swimming, walking) was provided. Those responding affirmatively, reported activity frequency from every/most days to < 2–3 times/month [29]. In this manuscript we define low activity frequency as < 1/week (i.e. those reporting activity as < 2–3 times/month and those reporting no ‘regular’ activity), hereafter referred to as inactivity. Cross-classifying binary measures at 33y and 50y gave two stable behaviour groups: ‘never inactive’ (active ≥1/week at both ages) and ‘persistently inactive’ (< 1/week at both ages); and two behaviour change groups: ‘deteriorating to inactivity’ (≥1/week at 33y, < 1/week at 50y) and ‘improving from inactivity’ (< 1/week at 33y, ≥1/week at 50y).

*Health and social factors at 33y and 50y:* include poor self-rated heath, adiposity, depression, low self-efficacy, manual SEP, not in paid employment, not married/co-habiting and parenthood at ≥2 children. Factors were identified from previous studies [8, 20], assessed prospectively at 33y and 50y, and, as in previous research [30], categorised as binary variables (details in Table 1). Similar to physical inactivity patterns 33y-to-50y, we identified two stable and two change groups 33y-to-50y, e.g. for SEP: always non-manual class, always manual class (i.e. stable groups) and upwardly mobile from manual to non-manual and downwardly mobile from non-manual to manual (i.e. change groups). For adiposity, we used obesity in analysis of baseline (33y) factors with subsequent inactivity 33y-to-50y; for analyses of simultaneous changes with inactivity 33y-to-50y we examined weight.

**Table 1:** Health and social factors at 33y and 50y^a^ and early-life covariates^b^, from the 1958 British birth cohort

	Age	Description	Categories/units	N(%) / Mean(SD)
Health and social factors at 33y and 50y (main exposures)				
Poor self-rated health	33y 50y	one question with 4 categories at 33y (5 categories at 50y) ranging from excellent to poor	dichotomised: ‘poor’ refers to two categories (‘fair’ and ‘poor’) combined	1444 (13.3) 1789 (18.4)
Obesity	33y 50y	body mass index (weight (kg) /height (m)^2^) ≥ 30 kg/m^2^	No; Yes	1227 (11.5) 2395 (25.6)
Depression	33y 50y	15 (yes/no) items at 33y (8 at 50y) from the psychological sub-scale of the Malaise Inventory	top (gender-specific) 10% defined as ‘depressed’^c^	1294 (11.8) 998 (10.4)
Low self-efficacy	33y 50y	3 (yes/no) questions: feel you get what you want out of life; have free choice and control over life; run life more or less as you want to. Items summed to create a score (0–3)	defined as scores 0–2	3036 (29.6) 2552 (26.6)
Manual SEP	33y 50y	categorized using the Registrar General’s Classification of occupations (if missing at 50y, 46y data used)	includes skilled manual and semiskilled/unskilled classes	4082 (40.1) 3063 (32.9)
Not in paid employment	33y 50y	derived from participant reports of their current main economic activity; includes unemployed, full time education/government training, temporary/permanently sick, looking after home/family, retired, other	No; Yes	2281 (20.8) 1489 (15.3)
Not married/co-habiting	33y 50y	not living with a lawful or live in partner, derived from household composition data	No; Yes	2263 (20.5) 1975 (20.3)
Parenthood (number of children)	33y 50y	all children (natural/adopted/partner’s/fostered) living in the household; identified from household composition data	dichotomised as ≥2 children living in the household	5600 (55.5) 3129 (32.1)
Early-life covariates				
Pre-pubertal stature	7y	measured by trained medical staff, to the nearest inch	cm	122.4 (5.9)
Hand control/ co-ordination problems	7y, 11y, 16y	at each age recorded as: no problems (score: 0); somewhat or certainly applies (score: 1); the three variables were summed across ages	Number of ages with problem: 0 (i.e. no problem at 7y, 11y and 16y), 1, 2, 3 (problems at 7y, 11y and 16y)	0: 6388 (57.9) 1: 3063 (27.8) 2: 1276 (11.6) 3: 308 (2.8)
Cognitive ability	16y	derived age standardised score for reading and mathematics tests and converted to 0–100 scale. Average of tests used (if missing, average from 11/7y used); converted to internally standardised z-scores.	NA^d^	NA^d^
SEP	birth	father’s occupation at birth (if missing at 7y); categorized using the Registrar General’s (1951) Classification	1. professional/managerial 2. skilled non-manual 3. skilled manual 4. semiskilled/unskilled/no male head	1: 2141 (18.0) 2: 1171 (9.9) 3: 5817 (48.9) 4: 2760 (23.2)
Household amenities	7y, 11y, 16y	three questions at each age on access to bathroom/indoor lavatory/hot water, scored as: sole use (0), shared (1), not available (2); the nine questions are summed across ages.	Score range: 0–18	1.07 (2.6)
Minimal parental education	birth, 7y	two questions on (i) mother and (ii) father having minimal schooling	No; Yes	6334 (60.1)
Parental divorce	33y	single question on parents ever permanently separating or divorced	No; Yes	1672 (15.4)

N varies dues to missing data

*SEP* Socio-economic position

^a^33y obesity is measured; all other 33y and 50y measures are self-reported

^b^Early-life covariates: measured (pre-pubertal stature), teacher reported (hand control/ co-ordination problems), tested (cognitive ability), parent-reported (social class, household amenities and parental education) or self-reported (parental divorce)

^c^see Pinto Pereira SM et al. *JAMA Psychiat*. 2014; 71 (12):1373–1380

^d^non-standardised values are not available because measures for the combination of ages are not meaningful

*Covariates* were identified from previous work [7] and recorded prospectively at birth (SEP), age 7y (pre-pubertal stature) and 16y (cognitive ability, parental education) or at multiple ages in childhood (hand control/co-ordination problems, household amenities) (details in Table 1).

### Statistical analysis

As a preliminary step, to identify factors for main analyses, we examined simple associations between factors and inactivity. These preliminary analyses assessed associations between each factor and inactivity concurrently for ages 33y and 50y separately (i.e. cross-sectionally) and for 33y factors with 50y inactivity. We then adjusted associations for all other factors at the same age. Any factor that was unrelated to inactivity at both 33 and 50y was excluded from further analyses; all other factors were included.

In main analyses to examine associations of factors at baseline (33y) with inactivity 33y-to-50y (objective i), we fit two multinomial logistic regression models, which provided Relative Risk Ratios (RRRs) and 95% confidence intervals (CIs). First, we compared persistently vs never inactive (i.e. most vs. least adverse behaviour) and deteriorating to inactivity vs never inactive (i.e. changing vs. remaining the same). Second, we compared improving from inactivity vs persistent inactivity. We then examined associations of factors 33y-to-50y and the four inactivity patterns over the same ages (objective ii), again using multinomial logistic regression. For these main analyses, gender differences in associations with inactivity were tested using an interaction term (gender*factor); there was little evidence of effect modification (Tables 2 and 3, footnotes), hence results are presented for genders combined. All associations were mutually adjusted for all other factors at the same life-stage, and then adjusted for early-life covariates.

**Table 2:** Associations (RRRs (95% CIs)) between baseline health and social factors (33y) and physical inactivity 33y-to-50y^a^

33y factor	Persistent vs. never inactive	Deteriorating vs. never inactive	Improving vs. persistently inactive
Poor self-rated health	2.01 (1.70,2.38)	1.38 (1.16,1.64)	0.77 (0.63,0.94)
Obesity	1.56 (1.30,1.86)	1.40 (1.18,1.67)	0.72 (0.57,0.90)
Depression	1.28 (1.05,1.57)	1.10 (0.89,1.36)	1.09 (0.88,1.35)
Low self-efficacy	1.41 (1.22,1.62)	1.19 (1.04,1.36)	0.86 (0.73,1.01)
Manual SEP	1.41 (1.24,1.61)	1.44 (1.28,1.63)	0.89 (0.76,1.04)
Not married/co-habiting	1.06 (0.91,1.24)	1.15 (0.98,1.35)	0.84 (0.69,1.02)
Parenthood (≥2 children)	1.11 (0.97,1.27)	1.03 (0.90,1.16)	1.15 (0.98,1.35)

Results from two multinomial logistic regression models: comparing (i) persistently vs never inactive and deteriorating to inactivity vs never inactive and (ii) improving from inactivity vs persistent inactivity

Models adjust for gender and for all factors in the table

p for gender interaction (in univariable analysis) ≥ 0.33 for all health and social factors (except *p* = 0.03, for parenthood)

^a^Physical inactivity N (%) at 33y: 3426 (31.3); at 50y: 2955 (30.4); % inactive 33–50y (averaged over 20 imputed datasets): Never inactive: 51.4; persistently inactive: 13.6; deteriorating: 17.2; improving: 17.9

*SEP* Socio-economic position

**Table 3:** Prevalence (%) and associations (RRRs (95% CIs)) between health and social factors (33y-to-50y) and physical inactivity 33y-to-50y

	%^a^	Persistent vs. never inactive	Deteriorating vs. never inactive	Improving vs. persistently inactive
Self-rated health				
Always good/excellent	73.8	ref	ref	ref
Improves	6.7	1.57 (1.23,2.01)	1.30 (1.04,1.62)	0.90 (0.67,1.21)
Worsens	12.6	1.98 (1.61,2.44)	2.20 (1.85,2.62)	0.61 (0.49,0.77)
Always poor	6.8	3.16 (2.51,3.99)	2.04 (1.58,2.64)	0.55 (0.42,0.71)
Weight change				
Stable (−5% to + 5%)	26.2	ref	ref	ref
Decrease (> − 5%)	11.0	1.22 (0.95,1.56)	1.04 (0.83,1.29)	0.91 (0.69,1.21)
Increase (> 5%)	62.8	1.31 (1.12,1.53)	1.26 (1.09,1.45)	0.82 (0.68,0.98)
Depression				
Never depressed	81.4	ref	ref	ref
Improves	7.5	1.12 (0.87,1.44)	0.92 (0.70,1.21)	1.17 (0.90,1.53)
Worsens	6.4	0.99 (0.76,1.29)	1.02 (0.80,1.30)	1.03 (0.77,1.39)
Always depressed	4.7	0.99 (0.70,1.38)	1.00 (0.73,1.37)	1.32 (0.95,1.84)
Self-efficacy				
Always high	58.1	ref	ref	ref
Became high	14.1	1.17 (0.96,1.42)	1.00 (0.83,1.19)	0.95 (0.76,1.20)
Worsening	12.4	1.60 (1.30,1.98)	1.22 (1.00,1.48)	0.77 (0.61,0.97)
Always low	15.4	1.88 (1.54,2.29)	1.39 (1.14,1.69)	0.73 (0.58,0.92)
SEP				
Always non-manual	48.6	ref	ref	ref
Upwardly mobile	14.5	1.23 (1.03,1.48)	1.26 (1.05,1.51)	0.95 (0.77,1.17)
Downwardly mobile	10.0	1.29 (1.03,1.63)	1.16 (0.92,1.47)	0.84 (0.64,1.10)
Always manual	26.9	1.49 (1.27,1.76)	1.49 (1.29,1.72)	0.86 (0.70,1.04)
Partnership				
Always partnered	68.2	ref	ref	ref
Gained partner	10.5	0.88 (0.70,1.10)	1.19 (0.98,1.43)	0.91 (0.68,1.22)
Lost partner	10.9	0.82 (0.64,1.05)	0.89 (0.74,1.07)	1.27 (0.96,1.66)
Always no partner	10.3	1.02 (0.82,1.27)	0.96 (0.77,1.20)	0.92 (0.71,1.19)
Parenthood (number of children)				
0/1 at both 33y & 50y	35.1	ref	ref	ref
Decreased number	33.5	1.05 (0.89,1.24)	1.03 (0.88,1.21)	1.19 (0.99,1.43)
Increased number	13.3	0.94 (0.76,1.16)	0.99 (0.81,1.21)	1.02 (0.79,1.30)
2+ at both 33y & 50y	18.1	1.10 (0.90,1.35)	0.93 (0.78,1.11)	1.16 (0.92,1.46)

Results from two multinomial logistic regression models: comparing (i) persistently vs never inactive and deteriorating to inactivity vs never inactive and (ii) improving from inactivity vs persistent inactivity

Models adjust for gender and for all factors in the table

p for gender interaction (in univariable analysis) ≥ 0.17 for all health and social factors

^a^Averaged over 20 imputed datasets

*SEP* Socio-economic position

We conducted sensitivity analyses to account for potential bi-directional associations of inactivity with adiposity or mental health [14, 15, 31, 32] and to control for previous activity level; i.e. we adjusted for 16y BMI and mental health and 23y activity.

To minimize data loss multiple imputation via chained equations was used to impute missing data on inactivity (11% at 33y; 21% at 50y), health and social factors (10% (33y marriage/co-habitation) to 24% (50y SEP)) and covariates (2% (16y cognition) to 30% (16y BMI)). Imputation models included all model variables, including previously identified key predictors of missingness (16y externalising and internalising behaviours, cognitive ability, and pre-pubertal stature) [28]. Regression analyses were run across 20 imputed datasets; overall estimates were attained using Rubin’s rules. Imputed results (presented here) were broadly similar to those using observed values.

## Results

A third of the population were inactive at 33y and 50y; between these ages, 35% changed their inactivity status with 17% deteriorating to inactivity and 18% improving from inactivity (Table 2, footnotes). Likewise, in this population, there were changes in health and social factors over the same ages; e.g. prevalence of poor health increased from 14 to 19% (Additional file 1: Table S1) with 13% worsening and 7% improving from poor health (Table 3). In preliminary analyses, most 33y and 50y health and social factors were associated with inactivity at either 33y or 50y (Additional file 1: Table S1) and therefore included in main analyses; an exception was not in paid employment, which was not analysed further.

### Baseline factors (33y) and inactivity 33y-to-50y

Poor health and obesity were associated with deteriorating to and improving from inactivity (Table 2); e.g. for poor health, RRRs_adjusted_ were 1.38(1.16,1.64) and 0.77(0.63,0.94) respectively. Low self-efficacy and manual SEP were associated with deteriorating to inactivity but not improving from inactivity. Baseline (33y) poor health, obesity, depression, low self-efficacy and manual SEP were all associated with persistent inactivity. Partnership and parenthood were not associated with inactivity patterns 33y-to-50y. Further adjustment for early-life factors attenuated associations, but most remained (Additional file 1: Table S2).

### Health and social factors 33y-to-50y and inactivity 33y-to-50y

Worsening (vs always good) health 33y-to-50y was associated with higher risk of deteriorating to inactivity 33y-to-50y (RRR_adjusted_:2.20(1.85,2.62)) and with lower risk of improving from inactivity (RRR_adjusted_:0.61(0.49,0.77); Table 3). However, improving health was also associated with deteriorating to inactivity (RRR_adjusted_:1.30(1.04,1.62)) but not with improving from inactivity. Weight gain was associated with higher risk of deteriorating to inactivity (RRR_adjusted_:1.26(1.09,1.45)) and lower risk of improving from inactivity (RRR_adjusted_:0.82(0.68,0.98)); there were no associations between weight loss and change in inactivity. Worsening self-efficacy was associated with a lower risk of improving from inactivity (RRR_adjusted_:0.77(0.61,0.97)); with a borderline association for deteriorating to inactivity. Downward social mobility was not associated with change in inactivity, but upward mobility was associated with higher risk of deteriorating to inactivity (RRR_adjusted_:1.26(1.05,1.51)). Remaining in poor health, low self-efficacy and manual SEP 33-to-50y were associated with elevated risk of deteriorating to inactivity (RRRs_adjusted_ ranged from 1.39 to 2.04) and, for all except manual SEP 33y-to-50y, with lower risk of improving from inactivity (RRRs_adjusted_ ranged from 0.55 to 0.73). Remaining in poor health, low self-efficacy and manual SEP 33-to-50y were also associated with elevated risk of inactivity persistence (RRR_adjusted_ ranged from 1.49 to 3.16). Adjustment for early-life factors attenuated associations, but most remained (Table 3 vs Additional file 1: Table S3). In sensitivity analyses (to account for potential bi-directional associations of inactivity with adiposity or mental health, and to control for previous activity level) including further adjustment for 16y BMI and mental health and 23y activity, showed that most associations remained, although attenuated slightly (Table 3 vs Additional file 1: Table S4). Finally, no associations were observed for depression, partnerships and parenthood 33y-to-50y with inactivity over the same ages.

## Discussion

In a general mid-life adult population, we found that poor-rated health and obesity at baseline (33y) were associated with subsequent change in inactivity 33y-to-50y. Moreover, *changes* in health and weight 33y-to-50y were associated with *changes* in inactivity over the same ages. To illustrate, worsening health (affecting 13% of the population) was associated with more than two-fold risk of deteriorating to inactivity and a 39% lower risk of improving from inactivity. Low self-efficacy at baseline was associated with subsequent deterioration to inactivity (but not improvement from inactivity), whereas worsening self-efficacy 33y-to-50y (affecting 12% of the population) was associated with a 23% lower risk of improving from inactivity. At baseline, manual SEP was associated with subsequent deterioration to inactivity (not with improvement from inactivity) but downward mobility 33y-to-50y was not associated with inactivity changes. As expected, we found that remaining in poor health, low self-efficacy and manual SEP 33-to-50y were associated with higher risk of inactivity persistence. Few associations were observed for depression, none of which were found for change in this factor. Finally, no associations were found for partnership or parenthood, for baseline or change measures. Employment status was unrelated to inactivity in mid-adulthood.

Study data are unique in respect of prospectively recording contemporary factors and inactivity at two ages almost 20y apart, with similar measures allowing examination of simultaneous changes. Also, several prospectively measured early-life covariates were available. Limitations are acknowledged. There is no consistent definition of inactivity; some studies use failure to meet recommended activity levels [3, 9], while others identify the least active in a population [33]. Our measure, based on self-reported infrequent leisure-time activity (< 1/week), is similar to that used by others [23–26], and has been found to be associated with mortality [24, 25]. However, misclassification of an individual’s inactivity status and corresponding pattern over time remains a possibility. Regarding health and social factors, our measures were extensive but not exhaustive, e.g. we could not consider changes in neighbourhood or intention to participate in activity, which may be important [34, 35]. For the considered factors and inactivity, the timing of transitions are unknown; e.g. some changes may have occurred immediately after 33y, whereas others may have been just before 50y. The study is observational and uncontrolled covariates, measurement error or bi-directional associations (e.g. with adiposity or mental health [14, 15, 31, 32]) cannot be excluded. However, adjustment for prior adiposity, mental health and physical activity had little effect on results. As with any long-term study, sample attrition occurred, but we maximised available data by including participants with an inactivity measure at either 33y or 50y and avoided sample reductions due to missing information by using multiple imputation.

Comparison with other studies is hampered by heterogeneity in inactivity measurement and, with few exceptions [36, 37], previous studies have focused on changes over shorter periods (i.e. months to a few years) [20] than our study. However, some consistencies can be identified. Approximately a third of our population were inactive in mid-adulthood, which aligns with a global estimate of 31% [38]; and prevalence of persistent inactivity 33y-to-50y in our population (14%) is broadly consistent with others [39]. It is noteworthy that the prevalence of some (e.g. poor health), but not all (e.g. partnership) factors varied with age, but, like inactivity, most showed changes, e.g. approximately 21% changed partnership status 33y-to-50y.

Notwithstanding previous research by others [17, 22, 33] and ourselves [7, 30, 40] on life-course influences on inactivity, our focus here addresses an identified knowledge gap on simultaneous changes in factors and inactivity [10, 20, 21]. The ages examined (33y and 50y) span mid-adulthood; if physical activity is at a low level at this life-stage, it is not easily increased later on [41]. In turn, physical activity at older ages has benefits e.g. for the prevention of onset and delayed progression of disability [42]. Thus, inactivity in mid-adulthood becomes increasingly important against the backdrop of an ageing population [43].

A major study finding, is that poor health and obesity at baseline as well as worsening health and weight gain 33y-to-50y were associated with higher risk of deteriorating to and lower risk of improving from inactivity. Notably, associations were generally robust to adjustment for concurrent and early-life factors. Such findings agree with the scant literature available [37, 39], e.g. showing, as expected, associations of greater weight increases with persistent inactivity vs never inactive [39]. Importantly, there was no evidence to support an effect of improving health or weight loss on improving from inactivity, suggesting that the barriers to and promoters of activity may not be equivalent. Such novel findings highlight the need to continually maintain health and weight to deter adverse activity patterns; this need is particularly important at the life-stage examined, as mid-adulthood is generally a time of declining health [44] and increasing adiposity [45].

For self-efficacy and depressive symptoms, our study adds to a limited literature. Self-efficacy could influence inactivity e.g. by lessening an individual’s perceived capability to overcome barriers to activity [16] and, while our measure is general rather than specific to activity, we found that low self-efficacy at baseline was associated with a 19% higher risk of deteriorating to inactivity. This finding in our general population sample, agrees with previous work among employees [46]. Further support for differences in the barriers to and promoters of activity is our novel finding that worsening self-efficacy 33y-to-50y was associated with 23% lower risk of improving from inactivity, although, improving self-efficacy 33y-to-50y was not associated with improvement from inactivity. For depressive symptoms, the null associations for simultaneous changes with inactivity, agree with previous reports in this cohort [15] and elsewhere [47]. These results suggest that depressive symptom levels do not exert a major influence on inactivity behaviour, at least in populations approaching their 50’s. However, other aspects of mental functioning, such as affective judgments (i.e. enjoyment and pleasure from activity) may predict inactivity change [20].

To our knowledge, simultaneous changes in SEP and inactivity have not been examined previously. As might be expected, we found consistently manual SEP 33y-to-50y to be associated with higher risk (by 49%) of persistent inactivity. However, associations were less consistent for simultaneous changes in SEP and inactivity. Changes in SEP were not associated with improvement from inactivity, but surprisingly, upward mobility 33y-to-50y was associated with a higher risk of deteriorating to inactivity. Thus, our findings extend the literature that has thus far examined SEP and activity at a particular age [17, 22, 37]. For parenthood (number of children in the household), we found no association between baseline status and subsequent inactivity or between simultaneous changes in parenthood and inactivity. These findings differ from a review of eight studies, predominantly in women [20], identifying motherhood as a predictor of deteriorating to inactivity, and another study [13], reporting that becoming a parent was associated with corresponding decreases in activity. Differences between findings could be due to our inclusion of men and women, the ages or time-frames considered (e.g. median follow-up of 3 years in the review vs almost two decades in our study) or differences in study design including inactivity definitions. In sum, our findings suggest that, over the long-term, parenthood has little impact on inactivity. Finally, for partnership status and employment, our null results contrast with cross-sectional studies [48, 49], but are consistent with a review of 16 and 14 studies respectively suggesting no associations with inactivity change [20]. Such observations are important because they highlight the limitations of previous studies that do not consider within-person inactivity changes over time.

## Conclusions

Longitudinal studies of factors related to inactivity change shed light on why some people initiate and maintain an active lifestyle and others do not. Our study adds new knowledge on likely contemporary influences on physical inactivity; it suggests that changes in inactivity status are not uncommon, and that health factors may be particularly important in relation to such changes. Associations for worsening of health factors with adverse inactivity changes did not always correspond to findings for improvement in health factors with improvement from inactivity. This finding, suggesting that barriers to activity may not be equivalent to promoters of activity, has implications for policies to encourage activity among the inactive [50]. Moreover, examining corresponding changes in factors and inactivity adds weight to our findings with regard to causality. Specifically, that mid-life poor health, weight gain and self-efficacy were associated with corresponding detrimental inactivity patterns. Findings highlight the importance of maintaining health, weight and self-efficacy across adulthood to deter adverse activity patterns. Several of these factors are relatively common, underscoring their potential importance for population levels of inactivity in mid-adulthood.

## Additional file

Additional file 1: **Table S1.** Prevalence of health and social factors at 33y and 50y and associations (ORs (95% CIs)) with physical inactivity at 33y or 50y. **Table S2.** Associations (RRRs (95% CIs)) between baseline health and social factors (33y) and physical inactivity 33y-to-50y. **Table S3.** Associations (RRRs (95% CIs)) between health and social factors (33y-to-50y) and physical inactivity 33y-to-50y. **Table S4.** Associations (RRRs (95% CIs)) between health and social factors (33y-to-50y) and physical inactivity 33y-to-50y (adjusted for additional covariates. (DOCX 33 kb)
