# Supplementary material for: Change in health and social factors in mid-adulthood and corresponding changes in leisure-time physical inactivity in a prospective cohort
Source: Int J Behav Nutr Phys Act. 2018 Sep 15;15:89. doi: 10.1186/s12966-018-0723-z (PMC6139142; doi:10.1186/s12966-018-0723-z)
Supplement: Supplementary file 1 — Table S1. Prevalence of health and social factors at 33y and 50y and associations (ORs (95% CIs)) with physical inactivity at 33y or 50y. Table S2. Associations (RRRs (95% CIs)) between baseline health and social factors (33y) and physical inactivity 33y-to-50y. Table S3. Associations (RRRs (95% CIs)) between health and social factors (33y-to-50y) and physical inactivity 33y-to-50y. Table S4. Associations (RRRs (95% CIs)) between health and social factors (33y-to-50y) and physical inactivity 33y-to-50y (adjusted for additional covariates. (DOCX 33 kb) [file 12966_2018_723_MOESM1_ESM.docx]

Additional file 1

Table S1 Prevalence of health and social factors at 33y and 50y and associations (ORs (95% CIs)) with physical inactivity at 33y or 50y

|  |  | 33y | | 50y | |
| --- | --- | --- | --- | --- | --- |
|  | %^¥^ | Unadjusted | Adjusted* | Unadjusted | Adjusted* |
| *33y factors* |  |  |  |  |  |
| Poor self-rated health | 13.5 | 1.83 (1.63,2.05) | 1.59 (1.41,1.81) | 1.64 (1.36,1.98) | 1.45 (1.27,1.65) |
| Obesity | 11.7 | 1.25 (1.10,1.41) | 1.18 (1.04,1.34) | 1.52 (1.33,1.73) | 1.43 (1.25,1.63) |
| Depression | 12.2 | 1.63 (1.44,1.84) | 1.31 (1.15,1.50) | 1.35 (1.17,1.55) | 1.07 (0.91,1.25) |
| Low self-efficacy | 29.5 | 1.43 (1.31,1.56) | 1.24 (1.13,1.36) | 1.40 (1.27,1.55) | 1.22 (1.10,1.36) |
| Manual SEP | 41.4 | 1.35 (1.24,1.47) | 1.20 (1.10,1.31) | 1.46 (1.33,1.60) | 1.35 (1.23,1.49) |
| Not in paid employment | 20.9 | 0.93 (0.84,1.03) | 0.91 (0.81,1.01) | 1.08 (0.95,1.23) | 0.94 (0.82,1.07) |
| Not married/co-habiting | 20.9 | 1.07 (0.97,1.18) | 0.93 (0.83,1.04) | 1.22 (1.09,1.36) | 1.15 (1.02,1.30) |
| Parenthood (≥2 children) | 51.6 | 1.26 (1.16,1.37) | 1.21 (1.10,1.32) | 1.01 (0.91,1.11) | 1.00 (0.90,1.11) |
| *50y factors* |  |  |  |  |  |
| Poor self-rated health | 19.4 |  |  | 2.31 (2.06,2.59) | 1.98 (1.74,2.25) |
| Obesity | 26.5 |  |  | 1.64 (1.49,1.79) | 1.47 (1.34,1.62) |
| Depression | 11.2 |  |  | 1.56 (1.36,1.80) | 1.01 (0.87,1.18) |
| Low self-efficacy | 27.8 |  |  | 1.68 (1.51,1.85) | 1.38 (1.23,1.54) |
| Manual SEP | 36.9 |  |  | 1.47 (1.33,1.62) | 1.28 (1.16,1.42) |
| Not in paid employment | 16.3 |  |  | 1.33 (1.19,1.49) | 0.88 (0.77,1.01) |
| Not married/co-habiting | 21.2 |  |  | 1.10 (0.99,1.22) | 0.92 (0.82,1.04) |
| Parenthood (≥2 children) | 31.4 |  |  | 0.90 (0.82,0.98) | 0.94 (0.85,1.04) |

SEP: socio-economic position

^¥^Averaged over 20 imputed datasets

Unadjusted: adjusted for gender only

*Additionally adjusted for all factors at the same life-stage

Table S2: Associations (RRRs (95% CIs)) between baseline health and social factors (33y) and physical inactivity 33y-to-50y^¥^

| 33y factor | Persistent  vs. never inactive | Deteriorating vs. never inactive | Improving vs. persistently inactive |
| --- | --- | --- | --- |
| Poor self-rated health | 1.87 (1.58,2.21) | 1.30 (1.09,1.55) | 0.79 (0.64,0.97) |
| Obesity | 1.48 (1.24,1.77) | 1.34 (1.13,1.60) | 0.73 (0.59,0.92) |
| Depression | 1.20 (0.98,1.47) | 1.04 (0.84,1.29) | 1.12 (0.90,1.38) |
| Low self-efficacy | 1.31 (1.14,1.51) | 1.13 (0.98,1.29) | 0.88 (0.75,1.04) |
| Manual SEP | 1.14 (0.99,1.32) | 1.21 (1.06,1.39) | 0.96 (0.81,1.13) |
| Not married/co-habiting | 1.02 (0.87,1.20) | 1.13 (0.96,1.32) | 0.85 (0.70,1.04) |
| ≥2 children | 1.08 (0.94,1.24) | 1.00 (0.88,1.14) | 1.16 (0.99,1.36) |

SEP: socio-economic position

^¥^Adjusted for gender, all factors in the table and early-life factors (SEP at birth, pre-pubertal stature, cognitive ability, parental education and divorce, hand control/co-ordination problems, household amenities)

Table S3: Associations (RRRs (95% CIs)) between health and social factors (33y-to-50y) and physical inactivity 33y-to-50y^¥^

|  | Persistent vs. never inactive | Deteriorating vs. never inactive | Improving vs. persistently inactive |
| --- | --- | --- | --- |
| Self-rated health |  |  |  |
| Always good/excellent | ref | ref | ref |
| Improves | 1.50 (1.17,1.92) | 1.25 (1.00,1.56) | 0.92 (0.69,1.23) |
| Worsens | 1.89 (1.54,2.33) | 2.12 (1.79,2.52) | 0.62 (0.50,0.78) |
| Always poor | 2.93 (2.32,3.71) | 1.92 (1.48,2.49) | 0.56 (0.43,0.73) |
| Weight change |  |  |  |
| Stable (-5% to +5%) | ref | ref | ref |
| Decrease (>-5%) | 1.21 (0.94,1.56) | 1.03 (0.83,1.29) | 0.91 (0.69,1.21) |
| Increase (>5%) | 1.29 (1.10,1.52) | 1.24 (1.07,1.44) | 0.82 (0.69,0.99) |
| Depression |  |  |  |
| Never depressed | ref | ref | ref |
| Improves | 1.07 (0.83,1.39) | 0.89 (0.68,1.16) | 1.18 (0.91,1.55) |
| Worsens | 0.98 (0.75,1.27) | 1.01 (0.79,1.30) | 1.04 (0.77,1.40) |
| Always depressed | 0.95 (0.67,1.33) | 0.97 (0.71,1.33) | 1.34 (0.96,1.87) |
| Self-efficacy |  |  |  |
| Always high | ref | ref | ref |
| Became high | 1.12 (0.93,1.37) | 0.96 (0.81,1.16) | 0.97 (0.77,1.21) |
| Worsening | 1.55 (1.26,1.91) | 1.19 (0.98,1.44) | 0.78 (0.61,0.98) |
| Always low | 1.76 (1.44,2.15) | 1.31 (1.07,1.61) | 0.75 (0.60,0.94) |
| SEP |  |  |  |
| Always non-manual | ref | ref | ref |
| Upwardly mobile | 1.08 (0.89,1.30) | 1.12 (0.93,1.35) | 0.99 (0.79,1.22) |
| Downwardly mobile | 1.18 (0.93,1.50) | 1.08 (0.85,1.36) | 0.86 (0.65,1.14) |
| Always manual | 1.22 (1.01,1.46) | 1.26 (1.07,1.48) | 0.91 (0.73,1.13) |
| Partner |  |  |  |
| Always partnered | ref | ref | ref |
| Gained partner | 0.87 (0.69,1.09) | 1.19 (0.98,1.43) | 0.91 (0.68,1.22) |
| Lost partner | 0.81 (0.63,1.04) | 0.89 (0.74,1.07) | 1.27 (0.97,1.66) |
| Always no partner | 0.99 (0.79,1.24) | 0.95 (0.76,1.19) | 0.93 (0.72,1.21) |
| Parenthood (N of children) |  |  |  |
| 0/1 at both 33y & 50y | ref | ref | ref |
| Decreased number | 1.03 (0.88,1.22) | 1.02 (0.86,1.19) | 1.19 (0.99,1.43) |
| Increased number | 0.97 (0.79,1.20) | 1.03 (0.85,1.26) | 1.00 (0.78,1.28) |
| 2+ at both 33y & 50y | 1.11 (0.90,1.36) | 0.94 (0.78,1.12) | 1.15 (0.92,1.45) |

SEP: socio-economic position

^¥^Adjusted for gender, all factors in the table and early-life factors (SEP at birth, pre-pubertal stature, cognitive ability, parental education and divorce, hand control/co-ordination problems, household amenities)

Table S4: Associations (RRRs (95% CIs)) between health and social factors (33y-to-50y) and physical inactivity 33y-to-50y (adjusted for additional covariates^¥^)

|  | Persistent vs. never inactive | Deteriorating vs. never inactive | Improving vs. persistently inactive |
| --- | --- | --- | --- |
| Self-rated health |  |  |  |
| Always good/excellent | ref | ref | ref |
| Improves | 1.42 (1.10,1.83) | 1.22 (0.97,1.52) | 0.93 (0.70,1.25) |
| Worsens | 1.86 (1.50,2.30) | 2.09 (1.75,2.49) | 0.63 (0.50,0.79) |
| Always poor | 2.72 (2.14,3.45) | 1.83 (1.40,2.38) | 0.59 (0.45,0.77) |
| Weight change |  |  |  |
| Stable (-5% to +5%) | ref | ref | ref |
| Decrease (>-5%) | 1.18 (0.91,1.53) | 1.01 (0.81,1.26) | 0.93 (0.70,1.23) |
| Increase (>5%) | 1.29 (1.10,1.53) | 1.25 (1.07,1.44) | 0.82 (0.68,0.99) |
| Depression |  |  |  |
| Never depressed | ref | ref | ref |
| Improves | 1.05 (0.81,1.35) | 0.88 (0.67,1.15) | 1.20 (0.92,1.57) |
| Worsens | 1.00 (0.76,1.30) | 1.03 (0.80,1.31) | 1.03 (0.77,1.39) |
| Always depressed | 0.94 (0.67,1.33) | 0.96 (0.70,1.33) | 1.34 (0.96,1.87) |
| Self-efficacy |  |  |  |
| Always high | ref | ref | ref |
| Became high | 1.10 (0.90,1.34) | 0.95 (0.79,1.14) | 0.97 (0.78,1.22) |
| Worsening | 1.48 (1.20,1.83) | 1.16 (0.96,1.41) | 0.79 (0.62,1.00) |
| Always low | 1.63 (1.33,2.00) | 1.26 (1.03,1.55) | 0.77 (0.61,0.96) |
| SEP |  |  |  |
| Always non-manual | ref | ref | ref |
| Upwardly mobile | 1.04 (0.86,1.26) | 1.11 (0.92,1.33) | 1.00 (0.81,1.24) |
| Downwardly mobile | 1.15 (0.91,1.46) | 1.07 (0.85,1.35) | 0.87 (0.66,1.15) |
| Always manual | 1.16 (0.96,1.40) | 1.23 (1.04,1.46) | 0.93 (0.75,1.15) |
| Partner |  |  |  |
| Always partnered | ref | ref | ref |
| Gained partner | 0.86 (0.68,1.08) | 1.18 (0.98,1.42) | 0.91 (0.68,1.23) |
| Lost partner | 0.81 (0.63,1.04) | 0.89 (0.74,1.08) | 1.27 (0.96,1.66) |
| Always no partner | 0.97 (0.77,1.22) | 0.94 (0.75,1.18) | 0.94 (0.72,1.22) |
| Parenthood (N of children) |  |  |  |
| 0/1 at both 33y & 50y | ref | ref | ref |
| Decreased number | 0.97 (0.82,1.14) | 0.98 (0.83,1.15) | 1.23 (1.02,1.47) |
| Increased number | 1.04 (0.84,1.29) | 1.06 (0.87,1.29) | 0.98 (0.76,1.26) |
| 2+ at both 33y & 50y | 1.09 (0.89,1.34) | 0.92 (0.77,1.11) | 1.16 (0.92,1.46) |

SEP: socio-economic position

^¥^Adjusted for gender, all factors in the table, early-life factors (SEP at birth, pre-pubertal stature, cognitive ability, parental education and divorce, hand control/co-ordination problems, household amenities) and additional covariates (16y BMI and mental health and 23y activity)
